# West Nile Virus Infection in American Robins: New Insights on Dose Response

**DOI:** 10.1371/journal.pone.0068537

**Published:** 2013-07-02

**Authors:** Kaci K. VanDalen, Jeffrey S. Hall, Larry Clark, Robert G. McLean, Cynthia Smeraski

**Affiliations:** 1 United States Department of Agriculture, Animal and Plant Health Inspection Service, Wildlife Services, National Wildlife Research Center, Fort Collins, Colorado, United States of America; 2 Colorado State University, Department of Biomedical Sciences, Fort Collins, Colorado, United States of America; University of Texas Medical Branch, United States of America

## Abstract

West Nile virus (WNV) is a vector-borne pathogen that was first detected in the United States in 1999. The natural transmission cycle of WNV involves mosquito vectors and avian hosts, which vary in their competency to transmit the virus. American robins are an abundant backyard species in the United States and appear to have an important role in the amplification and dissemination of WNV. In this study we examine the response of American robins to infection with various WNV doses within the range of those administered by some natural mosquito vectors. Thirty American robins were assigned a WNV dosage treatment and needle inoculated with 10^0.95^ PFU, 10^1.26^ PFU, 10^2.15^ PFU, or 10^3.15^ PFU. Serum samples were tested for the presence of infectious WNV and/or antibodies, while oral swabs were tested for the presence of WNV RNA. Five of the 30 (17%) robins had neutralizing antibodies to WNV prior to the experiment and none developed viremia or shed WNV RNA. The proportion of WNV-seronegative birds that became viremic after WNV inoculation increased in a dose dependent manner. At the lowest dose, only 40% (2/5) of the inoculated birds developed productive infections while at the highest dose, 100% (7/7) of the birds became viremic. Oral shedding of WNV RNA followed a similar trend where robins inoculated with the lower two doses were less likely to shed viral RNA (25%) than robins inoculated with one of the higher doses (92%). Viremia titers and morbidity did not increase in a dose dependent manner; only two birds succumbed to infection and, interestingly, both were inoculated with the lowest dose of WNV. It is clear that the disease ecology of WNV is a complex interplay of hosts, vectors, and viral dose delivered.

## Introduction

West Nile virus (WNV) was introduced into the United States at New York City in 1999 and spread rapidly across the continental United States and into Canada, Latin America, and the Caribbean within six years [1]. The natural transmission cycle of WNV involves mosquito vectors and avian hosts [2] and the Centers for Disease Control and Prevention have identified 326 avian species positive for WNV in their avian mortality database [3]. Avian hosts vary in their susceptibility to WNV infection. American crows ( *Corvus* *brachyrhynchos* ), blue jays ( *Cyanocitta* *cristata* ) and greater sage-grouse ( *Centrocercus* *urophasianus* ) experience near 100% mortality from experimental WNV infection [4-7], while disease severity in other avian species covers a broad spectrum [5,8,9].

Mosquitoes also vary in competency as vectors of WNV according to species and local populations as determined by their ability to become infected and in the quantity of virus that amplifies in their tissues [10,11]. The efficiency of mosquito infection increases with higher viremia titers in vertebrate hosts on which they feed [12]. Variation also exists in the amount of virus delivered by individual mosquitoes during feeding. For example, some *Culex* sp. expectorated anywhere from 10^0.78^-10^3.58^ plaque forming units (PFU) of virus during experimental feeding studies [12]. In another study, *Culex* *pipiens* *quinquefasciatus* transmitted an average of 10^4.3^ PFU of WNV but the amounts ranged from 10^0.5^-10^5.3^ PFU [13].

Host reservoir competency has been described as a function of titer and duration of viremia; the length of time a host has sufficient virus circulating in its blood to infect feeding mosquitoes [5]. However, an important aspect of susceptibility of a host has been overlooked in some studies. Since natural mosquito vectors deliver a broad range of virus doses during feeding, and hosts vary in their susceptibility, the response of the host to various viral doses is critical to understanding host reservoir competence and potential.

American robins ( *Turdus* *migratorius* ) are an abundant backyard species across North America, reside in proximity to humans, and are exposed to ornithophagous mosquitoes [14-19]. Robins are known to be a reservoir competent host for the closely related Flavivirus, St. Louis encephalitis virus (SLEV), and are epidemiologically important avian hosts for SLEV in the central United States [20,21]. Robins also appear to be reservoir competent hosts for WNV; experimental infection with high doses of WNV revealed relatively high viremia titers [5,22]. These viremia titers combined with their exposure to feeding mosquitoes suggest that American robins are likely to play an important role in the local disease ecology of WNV and its potential for spread to humans [16]. In this study, we examined the response of American robins to inoculation with various WNV doses within the range of those administered by natural *Culex* . sp. vectors with the goals of eliciting new insights regarding host reservoir competency, WNV transmission cycles, and human health risks.

## Materials and Methods

### Ethics Statement

All experiments were approved by the Institutional Animal Care and Use Committee of the United States Department of Agriculture, Animal and Plant Health Inspection Service, Wildlife Services, National Wildlife Research Center (NWRC), Fort Collins, CO, USA (Approval number NWRC QA-1276) and Colorado State University, Fort Collins, CO, USA (Approval number 05-160A June 21 2005). Adult American robins were collected at the Colorado State Forest Service, Colorado State University, Fort Collins, CO, USA in 2005 with verbal permission from the facility occupants. Robins were captured in mist nets under Colorado Scientific Collecting Permit Number 05-TR060 and United States Fish and Wildlife Services Federal permit number MB019065-1; no endangered or protected species were affected.

### Animals

Adult American robins were trapped in 2005, banded with unique identifying leg bands (National Band and Tag Company, Newport, KY, USA) and were housed in individual cages in a BSL-3 facility at Colorado State University, Fort Collins, CO, USA. They were fed a mixture of fruit, dog food and meal-worms and provided water ad libitum. Birds were weighed and observed daily to monitor their health status.

### WNV titration

WNV (NY99; provided by Centers for Disease Control and Prevention, Fort Collins, CO, USA) was diluted in a viral transport media, BA-1 (Hanks’ M-199 salts, 1% bovine serum albumin, 350 mg/L sodium bicarbonate, 100 U/mL penicillin, 100 mg/L streptomycin, and 1 mg/L of fungizone in 0.05 M Tris, pH 7.6), and titers were verified by plaque assay [23]. Briefly, the virus was serially diluted 10-fold with BA-1 through 10 ^-8^, and 100 µL of each dilution was added in duplicate to Vero cell (ATCC, Manassas, VA, USA) monolayers in six-well plates (Costar, Cambridge, MA, USA). After 1 h of incubation at 37°C, the cells were overlaid with 3 mL/well of 0.5% agarose in Minimum Essential Media (MEM; without phenol red) supplemented with 1% fetal bovine serum, 250 mg/L sodium bicarbonate, 29.2 mg/L l-glutamine, 1 mg/L fungizone, 100 units/mL penicillin, 100 mg/L streptomycin, pH 7.6. Two days later, cells were overlaid with 3 mL of 0.5% agarose in the supplemented MEM with 0.004% neutral red dye (Sigma Chemical Corp, St. Louis, MO, USA). Viral plaques were counted on 4 and 5 days post inoculation (dpi). The limit of detection of the virus plaque assay was 10^1.7^ PFU/mL.

### Inoculation and sampling

Robins were divided into cohorts and assigned a WNV treatment. Based on the methods described above, the four WNV treatments were titrated at 10^1.95^ PFU/mL 10^2.26^ PFU/mL, 10^3.15^ PFU/mL, and 10^4.15^ PFU/mL. Each treatment was administered subcutaneously in the inguinal fold in 0.1 mL total volume resulting in final dosages of 10^0.95^ PFU, 10^1.26^ PFU, 10^2.15^ PFU, and 10^3.15^ PFU, respectively. Birds were sampled daily from 0 - 9 dpi and again on 14 dpi. Blood (0.2 mL) was obtained from all robins by jugular puncture and serum was separated by centrifugation at 13,200 rpm for 5 min. Oral swabs were obtained using sterile cotton-tipped applicators and placed in vials containing 1.25 mL BA-1 [24]. Serum and swabs were stored at -80^°^C until analyses.

### WNV replication and shedding

Plaque assays (as described above) were performed on serum samples for quantification of WNV viremia [23]. Real-time reverse transcription polymerase chain reaction (RRT-PCR) was performed on oral swabs to detect oral shedding of WNV. Viral RNA was extracted from swabs using the QIAamp Viral RNA mini kit (Qiagen, Valencia, CA, USA) according to the manufacturer’s instructions. In an effort to concentrate small amounts of RNA, the eluted RNA (60 µL) was ethanol precipitated using standard procedures and re-suspended in 12 µL nuclease-free water. RRT-PCR was performed using Applied Biosystems TaqMan® One-step RT-PCR system (Life Technologies, Grand Island, NY, USA) and the protocol and primers described in Lanciotti et al., 2000 [25] on an ABI 7900HT (Life Technologies, Grand Island, NY, USA). We also followed the guidelines described in Lanciotti et al., 2000 [25] to determine positive samples (Ct values <37 in duplicate wells).

### Serology

Sera collected on 0- and 14 dpi were analyzed using an epitope-blocking Enzyme Linked ImmunoSorbent Assay (bELISA) [26] and 90% Plaque Reduction Neutralization Test (PRNT_90_) [23]. Two commercially available monoclonal antibodies (MAb) were used in the bELISA assays, MAb 6B6C-1 (specific for the genus Flavivirus E protein epitope) and MAb 3.1112G (specific for a WNV NS1 protein epitope). Samples with ≥ 30% inhibition in both bELISA assays were considered positive for WNV specific antibodies, while samples with ≥ 30% inhibition in the 6B6C-1 assay only were considered presumptive flavivirus (non-WNV) positive. Vero cells were used in the PRNT_90_ assay to detect neutralizing antibodies to WNV and its close flavivirus relative, SLEV. Serum samples were initially diluted 1:10 in BA-1 diluent and then 2-fold serially diluted through 10^-8^. Seventy-five microliters of each dilution was mixed with 75 µL of a known ChimeriVax^TM^-WNV or ChimeriVax^TM^-SLE preparation (Acambis Inc., Cambridge, MA, USA) in a polypropylene 96-well plate resulting in a starting 1:20 serum dilution. The virus-serum mixtures were incubated at 37°C for 1 h to allow for virus neutralization. These mixtures were then tested by plaque assay [23] as described above with the following modifications: ChimeriVax^TM^-WNV infected cells received a 2^nd^ overlay on 3 dpi and plaques were counted on 4 and 5 dpi, while ChimeriVax^TM^-SLE infected cells received a 2^nd^ overlay on 4 dpi and plaques were counted on 5 and 6 dpi. Specimens were considered positive for WNV neutralizing antibodies if they reduced plaque formation of ChimeriVax^TM^-WNV by at least 90% at a serum dilution 4-fold greater than ChimeriVax^TM^-SLE neutralization.

### Immunohistochemistry

Birds that survived infection were euthanized 14 dpi (two birds succumbed to WNV infection on 3dpi and 5 dpi). Four birds from each dosage cohort and the two negative control birds were perfused with 0.9% saline, followed by 4% paraformaldehyde (PFA) in 0.1M phosphate buffer. The brain, cervical spinal cord, and other major organs were sectioned for WNV antigen immunostaining to visualize WNV invasion of various tissues (Text S1).

## Results

### Pre-challenge serological status of American robins

Prior to inoculation with WNV, the serological status of each bird was determined. Both PRNT_90_ analysis and bELISAs revealed that 25 of the 30 birds had no serological evidence of previous WNV exposure, while five robins (17%) were positive for WNV antibodies by both PRNT_90_ and bELISA. Seven birds were positive by the bELISA (MAb 6B6C-1) but negative for anti-WNV antibodies. Because a previous flavivirus exposure could not be confirmed, these birds were included in the study as WNV seronegative.

### Morbidity and Mortality in WNV challenged American robins

Birds were monitored at least twice daily for overt signs of disease and general health status. Two seronegative birds, one inoculated with 10^0.95^ PFU and the other with 10^1.26^ PFU, died on 5 and 3 dpi, respectively. Daily weighing showed a slight (~2%) decline in body mass in birds infected with WNV versus those not infected (data not shown); no other obvious signs of disease were noted.

### Effects of WNV dose on viremia titers

**Figure 1:**
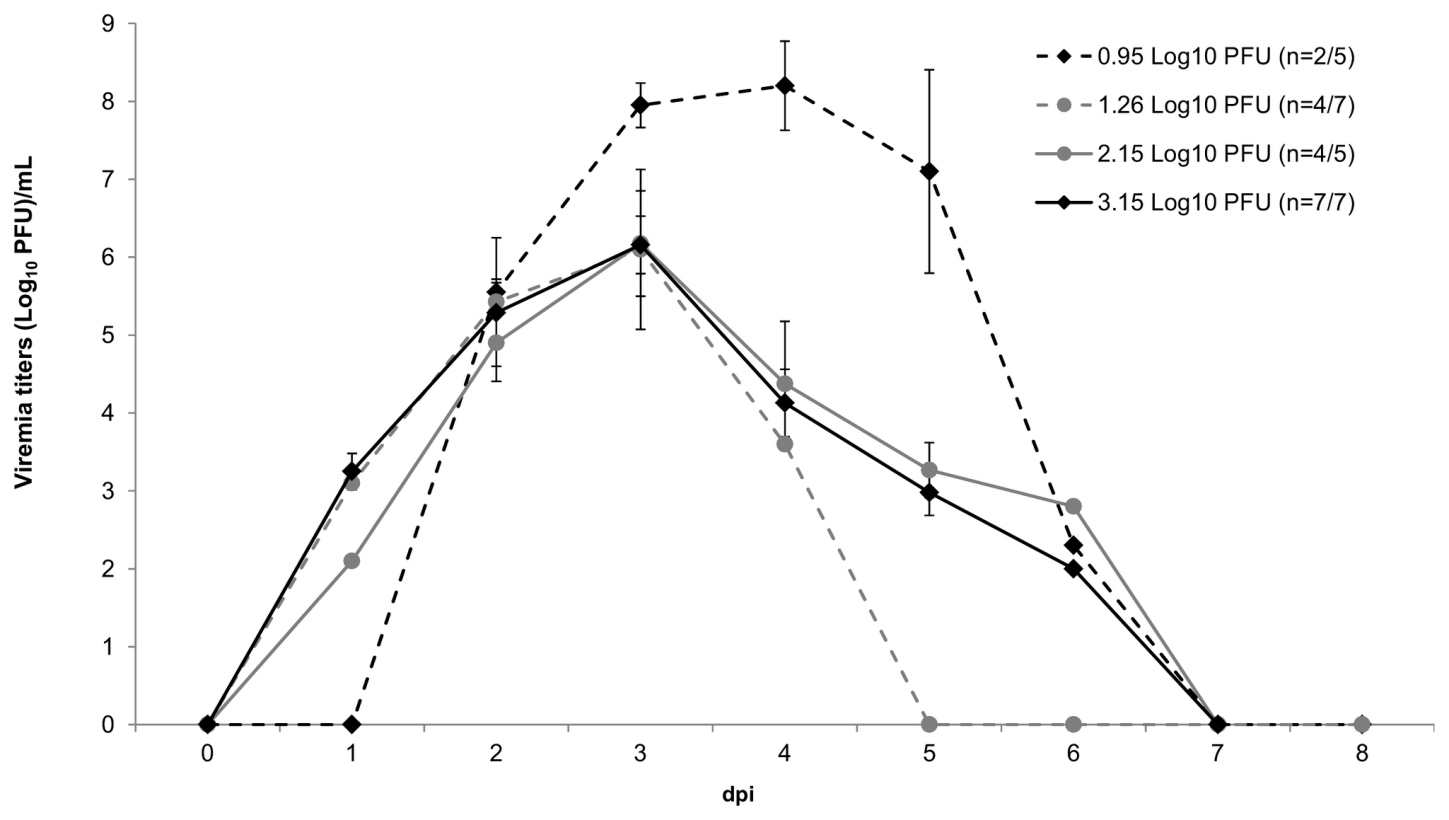
Mean viremia titers of WNV infected American robins. The number of individuals with detectable viremia compared to the number of individuals inoculated is indicated next to each dose in the legend. Titers were determined by plaque assay with 1.7 Log_10_ PFU/mL as the threshold of detection. Error bars represent standard error.

The percentage of WNV-seronegative birds that developed viremias versus the number inoculated was dose dependent (Table 1). Robins inoculated with the higher doses of WNV (10^2.15^ or 10^3.15^ PFU) were more likely to become viremic than robins inoculated with one of the lower doses of WNV (10^0.95^ or 10^1.26^ PFU; one-tailed Fisher’s exact test p < 0.05). Presumptive exposure to an undetermined flavivirus (based on the bELSISA MAb 6B6C-1) had no effect as 6/7 (86%) robins became viremic (Table 1). In the seventeen robins that developed viremia, peak viremia ranged from 10^3.6^ to 10^8.9^ PFU/mL. The two robins that became viremic at the lowest dose had remarkably higher mean viremias than the birds at the other three doses (Figure 1). The two robins that died during the experiment (ID # 404, 408) had the highest peak viremias (10^8.9^ PFU/mL, 5 dpi; 10^8.6^ PFU/mL, 3 dpi) and were inoculated with the smaller doses of virus (10^0.95^ and 10^1.26^ PFU, respectively). No viremia was detected in any bird after 6 dpi.

**Table 1:** Viremia titers in naive American robins experimentally infected with WNV as determined by plaque assay.

		**DPI**								
**Dose**	**Bird**	**0**	**1**	**2**	**3**	**4**	**5**	**6**
**10^0.95^ PFU**	407	−	−	−	−	−	−	−
	426	−	−	−	−	−	−	−
	404	−	−	5.7	8.3	8.9	8.7*	
	415	−	−	−	−	−	−	−
	418	−	−	5.4	7.6	7.5	5.5	2.3
**10^1.26^ PFU**	408	−	3.1	7.8	8.6*			
	403	−	−	3.6	3.2	−	−	−
	422	−	−	5.6	6.2	3.5	−	−
	419	−	−	−	−	−	−	−
	429	−	−	−	−	−	−	−
	416	−	−	−	−	−	−	−
	420^∞^	−	−	4.7	6.4	3.7	−	−
**10^2.15^ PFU**	428	−	−	3.4	6.7	5.4	2.5	−
	433^∞^	−	−	−	−	−	−	−
	434	−	−	5.0	7.1	4.9	3.8	−
	413^∞^	−	−	5.9	6.9	5.4	3.5	2.8
	423^∞^	−	2.1	5.3	4.0	1.8	−	−
**10^3.15^ PFU**	417	−	3.7	6.8	7.8	4.9	2.2	−
	410	−	−	3.3	6.3	6.4	4.1	2
	424	−	2.6	5.1	4.9	3.3	3.0	−
	425	−	3.4	5.7	6.9	3.8	2.9	−
	405^∞^	−	3.3	5.0	6.4	4.2	2.7	−
	401^∞^	−	2.5	4.6	5.1	3.0	−	−
	430^∞^	−	4.0	6.5	5.7	3.3	−	−

Titers expressed as Log**_10_** PFU/mL

∞ Presumptive positive flavivirus (non-WNV) exposure (bELISA MAb 6B6C-1), 0 dpi

* Bird died on the dpi indicated

- Titer below detection threshold of 1.7 Log_10_ PFU/mL

### Oral Shedding of WNV

The presence of WNV RNA in oral swabs from experimentally inoculated robins was typically first detected on 2 or 3 dpi (Table 2). Duration of shedding was variable and ranged from 2 to 14 dpi. Two of the birds continued to shed viral RNA on 14 dpi even though there was no detectable viremia at that point and all had successfully mounted neutralizing antibody responses. Similar to viremia data, oral shedding of WNV RNA was more likely to be detected in robins inoculated with the higher doses of WNV (10^2.15^ or 10^3.15^ PFU) than in robins inoculated with one of the lower doses of WNV (10^0.95^ or 10^1.26^ PFU; one-tailed Fisher’s exact test p < 0.05).

**Table 2:** Oral shedding in naive American robins experimentally infected with WNV as determined by RRT-PCR.

		**DPI**												
**Dose**	**Bird**	**0**	**1**	**2**	**3**	**4**	**5**	**6**	**7**	**8**	**9**	**14**
**10^0.95^ PFU**	407	−	−	−	−	−	−	−	−	−	−	−
	426	−	−	−	−	−	−	−	−	−	−	−
	404	−	−	−	**+**	**+**	+*					
	415	−	−	−	−	−	−	−	−	−	−	−
	418	−	−	−	−	−	−	−	−	−	−	−
**10^1.26^ PFU**	408	−	−	**+**	+*							
	403	−	−	**+**	−	−	−	−	−	−	−	−
	422	−	−	−	−	−	−	−	−	−	−	−
	419	−	−	−	−	−	−	−	−	−	−	−
	429	−	−	−	−	−	−	−	−	−	−	−
	416	−	−	−	−	−	−	−	−	−	−	−
	420^∞^	−	−	−	−	−	−	−	−	−	−	−
**10^2.15^ PFU**	428	−	−	**+**	**+**	**+**	**+**	**+**	**+**	**+**	**+**	**+**
	433^∞^	−	−	**+**	−	−	−	−	−	−	−	−
	434	−	−	−	**+**	**+**	−	−	−	−	−	−
	413^∞^	−	−	**+**	**+**	**+**	**+**	−	−	−	−	−
	423^∞^	−	−	−	**+**	−	**+**	**+**	−	−	−	−
**10^3.15^ PFU**	417	−	−	**+**	**+**	**+**	−	**+**	**+**	**+**	−	−
	410	−	−	−	−	**+**	−	**+**	**+**	−	−	**+**
	424	−	−	−	+	+	−	−	−	−	−	−
	425	−	−	−	−	**+**	**+**	**+**	**+**	**+**	**+**	**+**
	405^∞^	−	−	−	**+**	**+**	**+**	−	−	−	−	−
	401^∞^	−	−	−	−	−	−	−	−	−	−	−
	430^∞^	−	−	−	**+**	**+**	−	−	−	−	−	−

∞ Presumptive positive flavivirus (non-WNV) exposure (bELISA MAb 6B6C-1), 0 dpi

* Bird died on the dpi indicated

- No WNV RNA detected (Ct ≥ 37; or Undetermined)

+ WNV RNA detected (Ct < 37)

### Immune response of American robins after infection with WNV

**Figure 2:**
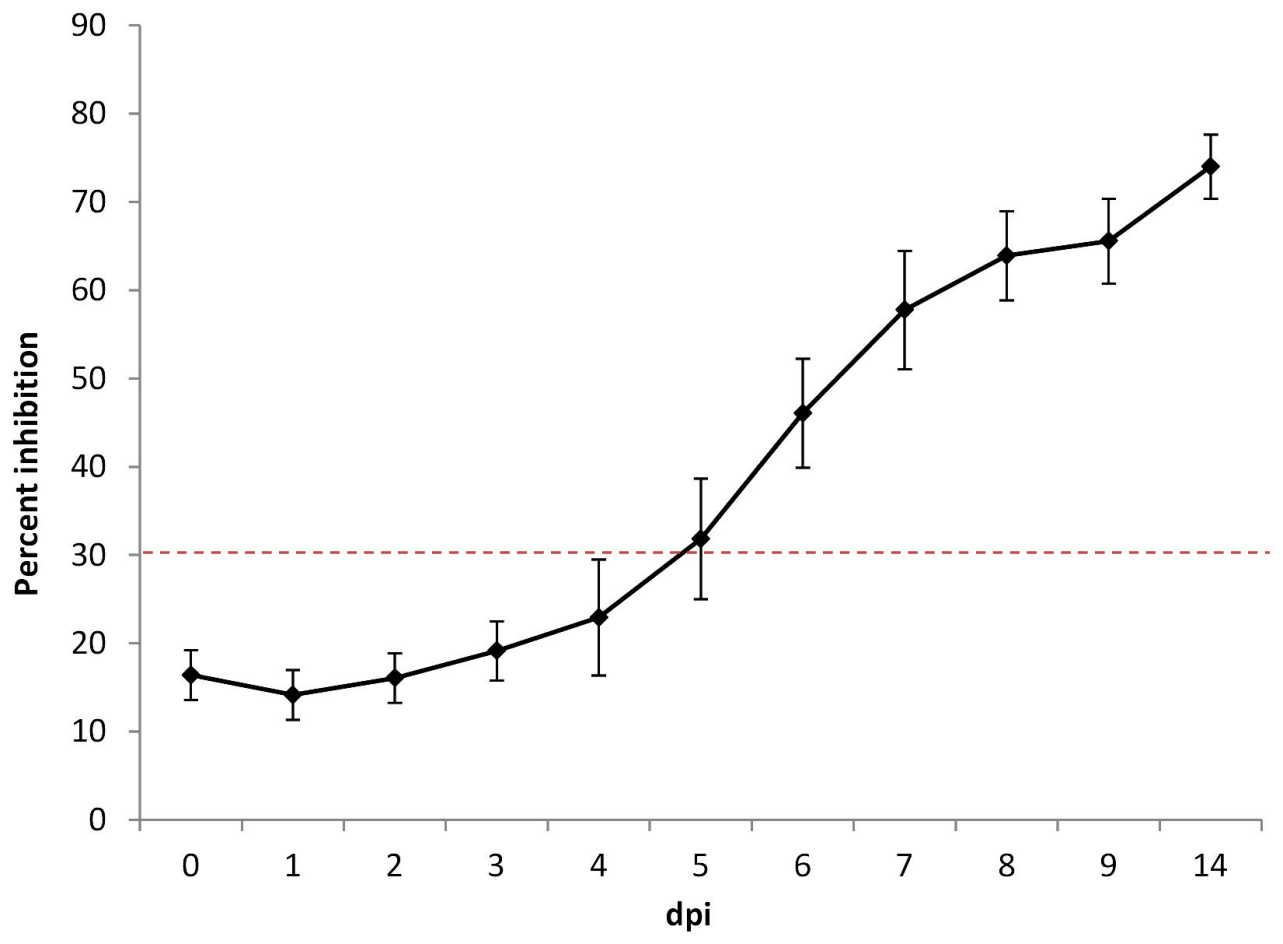
Mean antibody response of WNV infected American robins. Mean percent inhibition was determined by blocking ELISA with values ≥ 30% indicating a positive antibody response. Error bars represent standard error.

All birds that developed WNV viremia and/or shed WNV RNA (except the two birds that succumbed to infection at 3 and 5 dpi) developed antibodies to WNV. While the bELISA does not differentiate between various types of antibodies (i.e. IgM and IgG) it does give a reasonable insight into the temporal aspects of the host immune system’s activation and total antibody production. In this assay, 30% inhibition is considered the threshold of a positive antibody response; therefore anti-WNV antibodies were detectable in all robin sera, on average, by 5 dpi (Figure 2).

### WNV Seropositive Robins

Four WNV seropositive robins were challenged with two dosages of WNV (two each at 10^0.95^ PFU, 10^2.15^ PFU). No WNV seropositive robin inoculated with WNV developed detectable viremia or orally shed detectable levels of viral RNA. However, three birds challenged with WNV showed a substantial increase (≥ 4-fold) in neutralizing antibody titers versus pre-challenge titers, which is indicative of an anamnestic response (Table 3).

**Table 3:** WNV neutralizing antibody response (PRNT^90^) following WNV challenge of seropositive American robins.

**Dose (PFU)**	**Bird**	**0 dpi**	**14 dpi**
**0**	**421**	1:40	1:80
**10^0.95^**	**431**	1:320	1:320
**10^0.95^**	**172**	1:40	1:320
**10^2.15^**	**409**	1:320	1:1280
**10^2.15^**	**427**	1:320	1:5120

### Immunohistochemistry

The two robins (404,408) that died from experimental infection exhibited WNV immunolabeled cells in most major organ systems. In contrast, by 14 dpi in the remaining survivors of WNV infection, immunolabeling of WNV antigen was absent in the parenchyma of most organ systems except in goblet cells lining the villi and crypts in the upper intestine and ileum, and epithelia of the ureter branches (Text S1 Figure S1 Figure S2).

## Discussion

Recent studies on free-ranging avian hosts fed upon by vector mosquitoes detected through blood meal identification found that American robins were some of the most common and preferred food sources for many vector species [15-19]. *Culex* species are dominant vectors of WNV in North America [11,17,18,27,28] and based on analyses of blood meals from trapped mosquitoes and insect feeding shifts from avian to human sources, robins appear to have important role in the amplification and dissemination of the virus [16,27,28]. Because mosquitoes inject a broad range of viral doses during feeding [12,13], the measure of host susceptibility to WNV is more complex than can be determined by administering a single dose in experimental infection studies. Thus, an important component of any disease risk assessment is an evaluation of the host species response to various doses of the etiologic agent in question.

Our study shows that the proportion of American robins that became viremic increased in a dose dependent manner (40%, 57%, 80% and 100%, respectively). This result differs from previous dose response studies where 100% of house finches and mourning doves became viremic after inoculation with a very low WNV dose <0.3 log_10_ PFU [12]. Although robins inoculated with the higher doses of virus were more likely to become viremic, they did not develop higher viremia titers; a similar result to Reisen, et al. [12]. On average, the robins in our study had viremia curves that peaked later and at lower levels than those observed in an experimental study of two mosquito-inoculated American robins [5]. While the dose administered by the mosquitoes is unknown, the mosquito inoculation may have had enhancing effects on infection and viremia titers in the robins [29,30]. A more recent study inoculated two hatch-year American robins with 10^4^ PFU of WNV (strain 030019856 belonging to the WN02 clade) that resulted in viremia levels more similar to our findings in robins inoculated with 10^3.15^ PFU [22]. However, the hatch-year robins had viremia curves that peaked on 2 dpi, while the majority of robins in our study experienced viremias that peaked on 3 dpi. Field studies have indicated hatch-year birds as key amplifiers and transmitters of WNV [31] so the age of birds and/or the strain of WNV may have led to a quicker viremia response.

Another important factor of host reservoir competency is the development of host viremia titers infectious to feeding mosquitoes. Experimental studies of SLEV in Northern cardinals ( *Richmondena* *cardinalis* ) found *Cx* . *p* *. quinquefasciatus* mosquitoes that fed on birds with low viremias could still become infected, but the efficiency of mosquito infection increased to 80% with higher host viremias, 10^5.2^ PFU/mL [20]. A study using WNV showed a similar trend in different *Culex* sp. populations where as many as 90% became infected after feeding on blood with WNV concentrations of 10^6^-10^7^ PFU/mL [12]. In our study, 13/17 robins with detectable viremia (regardless of dose) experienced at least one day where the WNV viremia titer was at least 10^6.0^ PFU/mL. An additional three robins were sampled on at least one day with a viremia titer > 10^4.9^ PFU/mL, which is also considered infectious to several *Culex* sp. [10,32] (Figure 1). It appears that the likelihood of a robin developing viremia infectious to biting mosquitoes is not dependent on the dose of WNV inoculum given to the robins.

Our study also showed that oral shedding of WNV RNA from infected birds corresponded to dosage, only in terms of the number of birds shedding WNV RNA. While it was not feasible to quantitatively analyze the amount of WNV RNA detected in oral swabs, at the highest two doses (10^2.15^ PFU and 10^3.15^ PFU) 11/12 birds orally shed viral RNA with three still shedding on 14 dpi but at the lowest two doses only three birds had detectable viral RNA from oral swabs (Table 2). Our data indicate that although birds may clear infectious virus from their blood some may still shed viral RNA in oral secretions. This oral shedding may pose a risk to other birds, predators, and humans that handle them. Likewise, WNV antigen was still detected in some tissues two weeks after inoculation. Previous studies have demonstrated the presence of WNV RNA and in some instances, infectious WNV, in avian tissues for several weeks [33-35]. These results suggest that some avian hosts may be persistently infected and may even facilitate overwintering of the virus. We did not sample the robins past 14 dpi and did not attempt virus isolation from tissues so their susceptibility to persistent infection is still unclear. Because robins are an abundant competent reservoir host for WNV, future studies should investigate possibilities of persistent infection and potential overwintering.

Interestingly, one robin (433) inoculated with 10^2.15^ PFU of WNV, developed no detectable viremia, yet still shed viral RNA orally for 1 day and developed neutralizing antibodies to WNV. It is possible that the level and duration of viremia was missed with daily sampling. Another possibility for this bird’s muted response to WNV is that previous exposure to another flavivirus (as suggested by the bELISA MAb 6B6C-1) provided a slight protective response to WNV infection. Cross protection between flaviviruses has been documented previously. House finches infected with SLEV experienced reduced viremia titers when subsequently challenged with WNV. Similarly, house finches that survived initial infection with WNV produced no detectable viremia when subsequently challenged with SLEV [36].

Based on bELISA (MAb 3.112G) results, antibody response curves did not appear to differ with increasing doses of WNV. All seroconverting birds had essentially the same responses with antibody levels reaching the positive bELISA threshold of 30% at approximately 5-6 dpi (Figure 2). Four birds initially seropositive for WNV antibodies were challenged with WNV at two dosage levels. Three of these birds showed increases (≥ 4-fold) in antibody titers compared to their titers pre-inoculation (Table 3). The substantial increase in titer suggests an anamnestic response, which is commonly observed in subjects after challenge inoculation or booster vaccination. Separate studies that challenged WNV-seropositive house sparrows or house finches with WNV showed protective immunity and anamnestic responses similar to those in our study [36,37].

It is clear that the disease ecology of WNV is a complex interplay of factors including host species, vector species, and feeding behavior of the insect vectors. While the central role of American robins in WNV disease ecology has yet to be proven, certainly the abundance and proximity of robins to humans across the country, the apparent feeding choices of mosquito vectors, and the historical importance of robins in SLEV ecology, makes the reservoir host competence of robins to WNV important to describe. We show that the likelihood of American robins becoming viremic may be dependent on the dose of WNV administered by biting mosquitoes. Once a bird develops viremia, the titers produced are probably sufficient to infect feeding mosquitoes.

## Supporting Information

Figure S1 WNV antigen staining in tissues from robins that succumbed to WNV infection at 3 and 5 days post inoculation (dpi). Cross sections of organs are shown unless indicated otherwise. For each image, white fluorescent structures depict immunoreactive staining for WNV antigen and scale bars are 100 µm. A) characteristic clusters of WNV infected chromaffin and cortical cells in the adrenal gland, horizontal section, 5 dpi; inset, higher magnification, B) cardiac muscle fibers; 5 dpi; inset, higher magnification, C) splenic immune cells, 3 dpi, D) liver, 5 dpi; primarily Kupffer cells are stained, E) intestine, 3 dpi; Numerous cells of the crypts (both goblet and undifferentiated epithelial cells) were positive for WNV antigen. Villi in this portion of the duodenum have deteriorated (potentially from the high level of infection). However, WNV immunostaining was observed in sections more posterior, and was similar to Figure S2A, (m) surrounding smooth muscle. F and G) depict the intestinal wall of robins that died on 3 and 5 dpi, respectively. In these robins, WNV antigen staining was typically detected in muscle (or nerve) fibers along the medial wall (F), or throughout patches of the muscular coat (G), in blood vessels (arrow) supplying the intestine, and in scattered cells of the serosa (s). c, crypts (with virtually no antigen in these sections). H) pancreatic cells, 5 dpi. I) sparse immunolabelling in a horizontal section through the medullary cone (mc) and cortex (upper right) of the kidney, 3 dpi. Epithelial cells of branches of the ureter were also stained (see S2B). J) blood vessels (arrows) in stomach muscle, 3 dpi; inset, higher magnification of infected vascular endothelial cells.
(TIF)

Figure S2 Representative tissue sections of WNV antigen immunoreactivity from organs and the nervous system of robins that survived acute infection and those that succumbed during infection. For each image, white fluorescent structures depict immunoreactive staining for WNV antigen and scale bars are 100µm. A) Ileum of an asymptomatic robin two weeks after infection. Intestinal villi often exhibited WNV immunoreactivity in goblet cells in WNV exposed animals. Arrows point to the basal aspect of a few goblet cells, although there are numerous immunostained cells visible in this tissue. Mucin rich goblets (apical dark spheres) face the lumen of the intestine. B) Cross section through the kidney from a robin that survived WNV, illustrating WNV staining in a branch of the ureter. Ureteral epithelial cells were also immunostained in the 2 robins that did not survive infection. C) WNV immunopositive sympathetic neuron in the adrenal ganglion in the bird that died 5 dpi. Antigen was not present at 14 dpi in the adrenals and associated ganglia of robins that survived infection. D) Cross section through the brain showing the pineal gland (P) situated caudally between cerebral hemispheres. Arrow points to WNV antigen staining (at 3 dpi) in the leptomeninges (men) surrounding the brain. A few cells positive for WNV antigen were also observed along the pineal stalk in adjacent sections. E) Brain section through the choroid plexus (Cp) that projects into the ventricles. Choroidal epithelial cells (arrows) were immunolabeled by 3 dpi. F) Infected neurons in the hippocampus (Hp). WNV antigen staining was observed throughout the **cytoplasm, dendrites and axons** of neurons. Dotted line outlines the edge of the brain. Medial is left. AHP, **parahippocampal area**. G) Clusters of WNV infected cells in the brain at the ventral aspect of the habenular nucleus (Hb). Viral antigen is also dispersed through the **neuropil** among smaller infected **glial-like**/immune cells. v, lateral ventricle delineated by dotted line. H and I) Isolated neurons in the dorsal cerebellum (Cb) exhibit robust WNV immunostaining 14 dpi in an asymptomatic robin that survived infection. Dotted lines surround the adjacent hippocampus (Hp) in I. AHP, parahippocampal area. Medial is to the left.
(TIF)

Text S1 (DOC)
