# Supplementary figures and images for: West Nile Virus Infection in American Robins: New Insights on Dose Response
Source: PLoS One. 2013 Jul 2;8(7):e68537. doi: 10.1371/journal.pone.0068537 (PMC3699668; doi:10.1371/journal.pone.0068537)

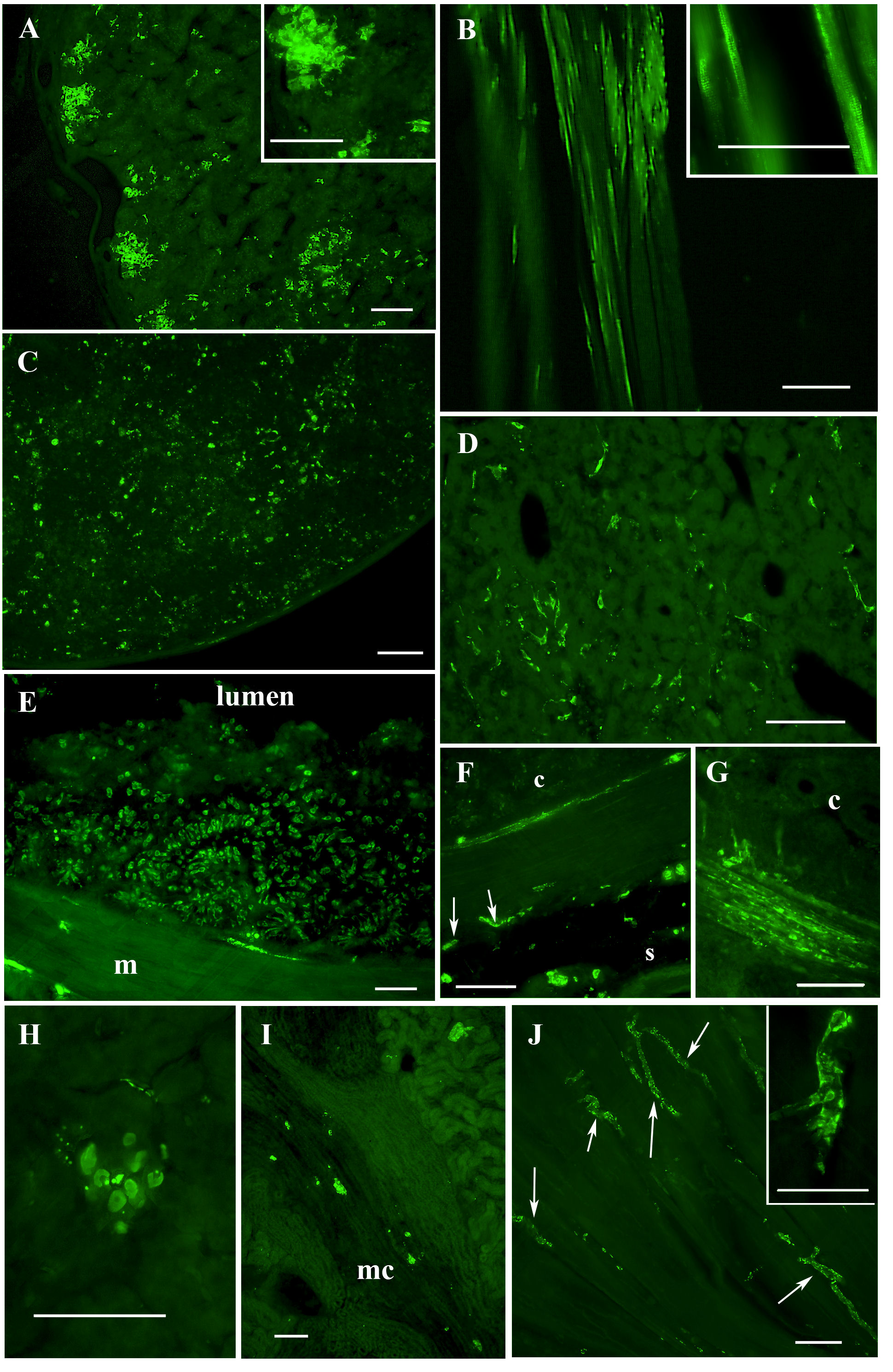

Supplement: Figure S1 — WNV antigen staining in tissues from robins that succumbed to WNV infection at 3 and 5 days post inoculation (dpi). Cross sections of organs are shown unless indicated otherwise. For each image, white fluorescent structures depict immunoreactive staining for WNV antigen and scale bars are 100 µm. A) characteristic clusters of WNV infected chromaffin and cortical cells in the adrenal gland, horizontal section, 5 dpi; inset, higher magnification, B) cardiac muscle fibers; 5 dpi; inset, higher magnification, C) splenic immune cells, 3 dpi, D) liver, 5 dpi; primarily Kupffer cells are stained, E) intestine, 3 dpi; Numerous cells of the crypts (both goblet and undifferentiated epithelial cells) were positive for WNV antigen. Villi in this portion of the duodenum have deteriorated (potentially from the high level of infection). However, WNV immunostaining was observed in sections more posterior, and was similar to Figure S2A, (m) surrounding smooth muscle. F and G) depict the intestinal wall of robins that died on 3 and 5 dpi, respectively. In these robins, WNV antigen staining was typically detected in muscle (or nerve) fibers along the medial wall (F), or throughout patches of the muscular coat (G), in blood vessels (arrow) supplying the intestine, and in scattered cells of the serosa (s). c, crypts (with virtually no antigen in these sections). H) pancreatic cells, 5 dpi. I) sparse immunolabelling in a horizontal section through the medullary cone (mc) and cortex (upper right) of the kidney, 3 dpi. Epithelial cells of branches of the ureter were also stained (see S2B). J) blood vessels (arrows) in stomach muscle, 3 dpi; inset, higher magnification of infected vascular endothelial cells. (TIF) [file pone.0068537.s001.tif]

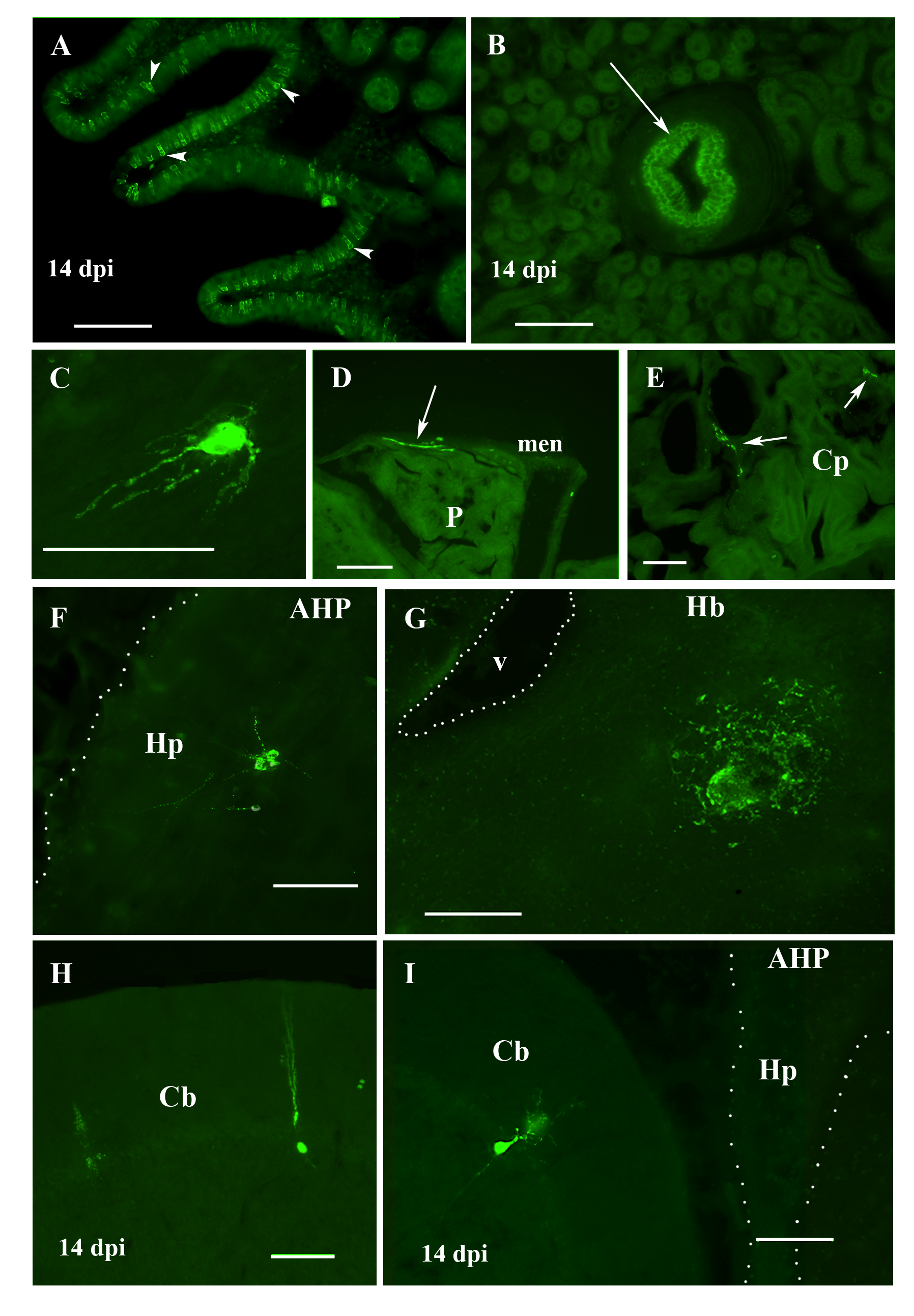

Supplement: Figure S2 — Representative tissue sections of WNV antigen immunoreactivity from organs and the nervous system of robins that survived acute infection and those that succumbed during infection. For each image, white fluorescent structures depict immunoreactive staining for WNV antigen and scale bars are 100µm. A) Ileum of an asymptomatic robin two weeks after infection. Intestinal villi often exhibited WNV immunoreactivity in goblet cells in WNV exposed animals. Arrows point to the basal aspect of a few goblet cells, although there are numerous immunostained cells visible in this tissue. Mucin rich goblets (apical dark spheres) face the lumen of the intestine. B) Cross section through the kidney from a robin that survived WNV, illustrating WNV staining in a branch of the ureter. Ureteral epithelial cells were also immunostained in the 2 robins that did not survive infection. C) WNV immunopositive sympathetic neuron in the adrenal ganglion in the bird that died 5 dpi. Antigen was not present at 14 dpi in the adrenals and associated ganglia of robins that survived infection. D) Cross section through the brain showing the pineal gland (P) situated caudally between cerebral hemispheres. Arrow points to WNV antigen staining (at 3 dpi) in the leptomeninges (men) surrounding the brain. A few cells positive for WNV antigen were also observed along the pineal stalk in adjacent sections. E) Brain section through the choroid plexus (Cp) that projects into the ventricles. Choroidal epithelial cells (arrows) were immunolabeled by 3 dpi. F) Infected neurons in the hippocampus (Hp). WNV antigen staining was observed throughout the cytoplasm, dendrites and axons of neurons. Dotted line outlines the edge of the brain. Medial is left. AHP, parahippocampal area. G) Clusters of WNV infected cells in the brain at the ventral aspect of the habenular nucleus (Hb). Viral antigen is also dispersed through the neuropil among smaller infected glial-like/immune cells. v, lateral ventricle delineated by dot [file pone.0068537.s002.tif]
