# Supplementary material for: West Nile Virus Infection in American Robins: New Insights on Dose Response
Source: PLoS One. 2013 Jul 2;8(7):e68537. doi: 10.1371/journal.pone.0068537 (PMC3699668; doi:10.1371/journal.pone.0068537)
Supplement: Text S1 — (DOC) [file pone.0068537.s003.doc]

**Text S1 Immunohistochemistry**

Four birds from each dosage cohort and the two control birds were euthanized and perfused with 0.9% saline, followed by 4% paraformaldehyde (PFA) in 0.1M phosphate buffer. The brain, cervical spinal cord, and major organs (heart, liver, spleen, proventriculus, stomach, intestine [duodenum and ileum], pancrease, adrenal glands, kidney, and gonads) were sampled and cryoprotected (25% sucrose in 4%PFA) for frozen serial sectioning at 35- 40 μm. The entire brain was serially sectioned, and every fourth section was processed to localize WNV antigen. Sections from the other organs (5 sections/700 μm, 15-25 sections/organ/bird) were similarly obtained. Small organs such as the adrenal gland, gonad and rostral aspect of the cervical spinal cord were sectioned in entirety.

Tissue sections were incubated for one hour in blocking solution [0.1M phosphate buffered saline (PBS), 4% normal goat serum, 0.4% Triton X-100, 1% bovine serum albumin (BSA)]. Sections were transferred to primary antibody solution containing WNV mouse hyperimmune polyclonal antibody (1:1000, CDC in PBS, 1% normal goat serum, 0.4% Triton X-100, 1% BSA) and incubated at room temperature overnight. Following several PBS rinses, WNV-protein staining was visualized using goat anti-mouse immunoglobulin G conjugated to either Alexa Fluor 488 (green) or Alexa Fluor 594 (red) diluted 1:400 in PBS, 1% BSA, 0.02% Triton X-100. Omitting the primary or secondary antibody, or substituting primary antibody with normal mouse ascites fluid eliminated WNV immunostaining in tissues.

Mounted tissues were examined using an epifluorescent Nikon Eclipse E800 microscope equipped with appropriate filters (FITC, TRITC) and a CCD camera (Spot RTKE-slider, Diagnostic Instruments) for capturing digital fluorescent and light images. ImageProPlus or Adobe Photoshop was used to create composites of images and figures.

Tissue from the two robins (404, 408) that died from experimental infection exhibited WNV immunolabeled cells in most major organ systems. Both birds exhibited WNV antigen in similar structures/cells of each organ. WNV antigen was observed in clusters of cells within the chromaffin islets and cords of adrenocortical tissues, as well as in neurons within the adrenal ganglia (Figures S1 and S2). Prominent WNV antigen was evident in muscle fibers and epicardium of the heart, distributed throughout the spleen, and in Kupffer cells of the liver. Within the intestine, light WNV immunostaining was observed in villi epithelia, and in intestinal crypts, and was restricted to the basal portion of goblet cells. More intense staining occurred in the surrounding muscular layer and serosa of the intestine. Focal clusters of pancreatic cells exhibited WNV immunostaining as well as diffuse interstromal macrophage-like cells. Scattered cells of both the renal medulla and cortex were positive for WNV antigen along with epithelial cells lining the lumen of secondary ureter branches. Immunostaining detected in the ureteral branches of the kidney was less intense than that observed in the medullary cones and cortex. Frequently, labeled antigen was observed in endothelial cells of blood (or lymphatic) vessels supplying the organs and in the serosa. WNV positive cells were observed scattered interstitially in the ovary and testis, however, the parenchyma of the proventriculus and stomach (ventriculus) were essentially free of viral antigen. Figure S1 illustrates immunolabelled viral antigen in representative sections of tissue from robins that died from WNV infection.

By 14 dpi, immunolabeling of WNV antigen was absent in the parenchyma of most organ systems from survivors of WNV infection. Two exceptions were goblet cells lining the villi and crypts in the upper intestine and ileum, and epithelia of the ureter branches. In these robins, levels of WNV immunostaining appeared unrelated to WNV dose, antibody status prior to inoculation, or levels of viremia. Half of the robins sampled that survived WNV infection (i.e., 7/14 sampled survivors) exhibited moderate to high levels of WNV immunolabel in the basal aspect of goblet cells of the intestine, located more often along the sides of intestinal villi than in the crypts. A representative example of WNV antigen in goblet cells is shown in figure S2A. Presence of WNV labeled antigen in the crypts often occurred in diffuse patches of goblet cells, although in 2 birds, labelling throughout most of the crypts was observed, as in one of the robins that succumbed to infection (Figure S1E). In contrast, faintly stained outlines of WNV immunoreactive goblet cells were observed in intestinal sections of the two control robins. This may be indicative of residual infection from past exposure to flavivirus. For example, one control bird, that had pre-existing antibodies specific to WNV (#421), exhibited numerous lightly stained goblet cells throughout many intestinal sections. The other control robin, seronegative for WNV specific and general flavivirus antibodies, exhibited only a few patches of immunolabeled goblet-like cells. This weak level of immunostaining in the intestine was also observed in the seven other WNV-inoculated robins examined, 4 of which developed peak viremia greater than 10^5^ PFU/mL, 3 were nonviremic. In the kidney, light immunoreactivity was restricted to epithelial cells in some secondary branches of the ureter (Figure S2B) in all 14 surviving robins (WNV inoculated) sampled and one of the control robins (with pre-existing antibodies specific to WNV). Medullary and cortical epithelial cells in the kidney were free of viral antigen. Staining of intestinal goblet cells or epithelial cells of ureteral branches was not observed in sections incubated in normal mouse ascites fluid in place of the WNV mouse polyclonal antibody.

Staining of WNV in the nervous system was limited in robins that succumbed to experimental infection at 3 and 5 dpi. By 3 dpi, little viral staining was observed in the brain parenchyma, however infected cells were observed sporadically in pituitary, leptomeninges that surround the brain, the choroid plexus, and pineal gland tissues (Figure S2). At the rostral aspect of the brain, where the olfactory nerve joins the olfactory bulb, the surrounding connective tissue and associated blood vessels were scattered with WNV immunolabeled cells, but the olfactory bulb parenchyma was free from virus. A single cluster of stained cells adjacent to a blood vessel was observed in the optic tectum. Slightly more viral antigen was detected in the brain of the robin that succumbed to WNV infection at 5 dpi. Again, a small number of cells within the choroid plexus, pituitary, and pineal were WNV immunostained. In addition, a few isolated neurons in the hippocampus and mesopallium were labeled. Viral antigen was unilaterally observed at the base of the habenula as punctate immunolabel dispersed within the brain parenchyma, as well as within small glial-like cells. Clusters of cells exhibiting WNV immunolabel were also located between or on the edge of the cerebellum lobes, and likely represent cells associated with the leptomeninges or choroid plexus that cover the lobes. Some small cells associated with the cerebrovasculature were also detected, although sporadically throughout the brain. The cervical spinal cord was free of virus in both birds.

In the central nervous system of surviving and control robins, WNV antigen was absent in the parenchyma of the brain, pituitary, pineal gland, cervical spinal cord, leptomeninges, and choroid plexus. Occasional WNV label of small capillaries of the cerebrovasculature was observed in two robins with peak viremia of 10^5^ PFU/mL. A few cerebellar cells (S2H,I) were also distinctly immunolabeled in the robin with the highest peak viremia among the survivors (10^7.5^ PFU/mL).
